# Triazolobithiophene Light Absorbing Self-Assembled Monolayers: Synthesis and Mass Spectrometry Applications

**DOI:** 10.3390/molecules16108758

**Published:** 2011-10-19

**Authors:** Ghislain Tsague Kenfack, Andreas Schinkovitz, Suresh Babu, Kamal Elouarzaki, Marylène Dias, Séverine Derbré, Jean-Jacques Helesbeux, Eric Levillain, Pascal Richomme, Denis Séraphin

**Affiliations:** 1 Laboratoire SONAS, Université d’Angers, IFR 149 QUASAV, 16 bd Daviers, 49100, Angers, France; 2 Laboratoire MOLTECH-Anjou, Université d'Angers-CNRS, UMR 6200 du CNRS, 2 bd Lavoisier, 49045, Angers, France

**Keywords:** light absorbing SAMs, DIAMS MS

## Abstract

The synthesis of five light absorbing triazolobithiophenic thiols, which were utilized for producing self-assembled monolayers (SAMs) on gold surfaces, is presented. The monolayer formation was monitored by cyclic voltammetry, indicating excellent surface coverage. The new triazolobithiophenic compounds exhibited an absorption maximum around 340 nm, which is close to the emission wavelength of a standard nitrogen laser. Consequently these compounds could be used to aid ionization in laser desorption mass spectrometry (MS).

## 1. Introduction

Light absorbing self-assembled monolayers (SAMs) offer very interesting properties for various scientific and technical applications [1,2,3,4,5,6]. Some of the latter are photo-switch systems [5,7], light detectors [8], and light to current/energy convertors [1,9,10,11]. Furthermore, SAMs have been proposed to aid ionization in laser desorption MS. On the one hand, Mrksich combined self-assembled monolayers with matrix-assisted laser desorption/ionization mass spectrometry (SAMDI mass spectrometry) that permitted characterization of functionalized thiolates anchored to gold surfaces as a monolayer. Such a technique is therefore useful for characterizing chemical and biochemical interactions at surfaces [12,13,14]. On the other hand, SAM’s also help characterization by mass spectrometry of analytes that do not chemically interact with the monolayer [2,3,4].

**Figure 1:**
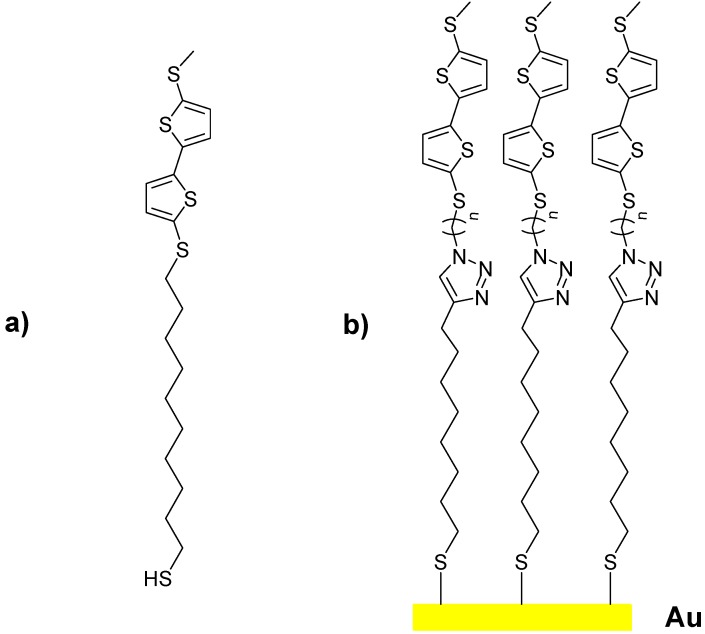
(**a**) 10-(5'-(methylthio)-2,2'-bithiophen-5-ylthio)decane-1-thiol; (**b**) triazolo-5,5'-disubstituted-2,2’-bithiophene immobilized on a gold surface as SAMs.

With respect to the intended application, various light absorbing systems such as fullerenes [5], diphenyldiacetylene [7], and porphyrin derivatives [8,9,10,11] have been described. Among conjugated polymers, oligothiophenes have been extensively studied over the past decades due to interest in their applicability in the field of molecular electronics and linear or branched oligothiophene SAMs have been reported [15,16,17]. Despite these comprehensive studies, hardly anything is known about SAMs utilizing bithiophenes [18,19] as photo active groups [3]. 10-(5'-(Methylthio)-2,2'-bithiophen-5-ylthio)decane-1-thiol [1, Figure 1a] has been used to aid ionization in the recently developed “desorption/ionization on self-assembled monolayer surfaces” (DIAMS) method [3]. In continuation of this work, five triazolobitiophenic SAMs of general structure **b** [Figure 1b] have been produced. Exhibiting enhanced surface coverage and long-term stability, these SAMs further represent ideal candidates for advanced studies of the light to energy transfer during MALDI-TOF MS recordings. Indeed, earlier experiments have shown that the *S*-acetylated derivative of **1** [Figure 1a] exhibits an absorption maximum (λmax) of 340 nm, which is close to the emission wavelength of a standard nitrogen laser (337 nm) [3]. These specific absorption properties are primarily due to the bithiophenic moiety. With respect to the DIAMS application these properties were preserved in all the new molecules.

Apart from these selective features, surface coverage together with long term stability represent crucial parameters for the quality of SAMs. The most basic bithiophenic SAM was produced by attaching a bithiophene moiety to a –(CH_2_)_n_–SH side chain of desired length, as previously described [3]. To increase coverage and stability, bithiophenic SAMs can be modified either at the terminal position or within the linker chain. It is well known that the functional group located on the surface of the monolayer, or in subterminal position significantly impacts packing arrangement. Indeed, van der Waals forces [20,21,22], dipole-dipole interactions [23,24,25], steric effect [26], hydrogen bonding [27,28,29,30,31,32,33,34,35] and π-π stacking [36] are all important factors for SAMs organization and stability. As changing the methyl group at the surface of SAMs requires a synthetic strategy with a complex desymmetrisation of the initial bithiophene, the authors decided to explore the effect of an extra aromatic ring in the subterminal part of the monolayer. In this context, the introduction of a 1,2,3-triazole ring appeared to be a good starting point, because the synthesis of the corresponding triazolobithiophene derivative could be easily achieved by a convergent click reaction. Further, the aromatic system (1,2,3-triazole ring) may exhibit π-π stacking [37,38] which could improve stability of the monolayers.

**Scheme 1:**
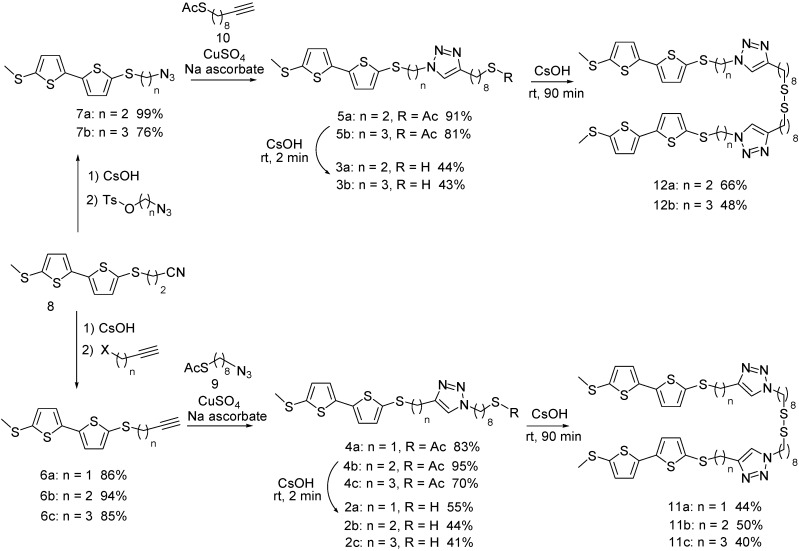
Synthesis of triazolobithiophenes **2a**–**c** and **3a**,**b**.

Preparations of self-assembled monolayers bearing a 1,2,3-triazole by applying a direct or a post-functionalization strategy have been previously reported [39,40], but this is the first report concerning triazolobithiophenic SAMs. The current manuscript presents the synthesis of thiols **2a**–**c** and **3a**,**b** as well as their corresponding disulfide derivatives (Scheme 1). Subsequently free and immobilized molecules were studied by electrochemistry, and a potential application for this new group of light absorbing SAMs will be presented.

## 2. Results and Discussion

The Huisgen reaction [41] consists of a 1,3-dipolar cycloaddition between a terminal alkyne and an organic azide, enabling the formation of mixture of 1,4 and 1,5-disubstituted triazole isomers [42]. Based on this approach, Sharpless *et al.* [43] developed the concept of click chemistry, which has become a highly recognized and frequently performed reaction in several fields of applied chemistry, such as biological, polymer and materials sciences, as well as drug discovery. Consequently the reaction appeared to be suitable for the design of bithiophenic SAMs. Considering that the location of the triazole moiety within the subterminal part of the monolayer might influence its stability [36,37], several different triazolobithiophenic derivatives were synthesized (Scheme 1). One group displayed either one, two or three carbons between the triazole ring and the bithiophene moiety (Scheme 1, triazolobithiophenes **4a**–**c**), while exhibiting a N–C connection between the triazole ring and the aliphatic side chain. Alternatively, a second group of triazolobithiophenes displaying a C–C connection between the triazole and the (CH_2_)_8_-SCOCH_3_ moiety were created (Scheme 1, triazolobithiophenes **5a**,**b**). Alkynylbithiophenes **6a**–**c** and azidoalkylbithiophenes **7a**,**b** were synthesized from 3-(5'-(methylthio)-2,2'-bithiophen-5-ylthio)propanenitrile following a strategy proposed by Becher *et al.* [3,43] and underwent a copper (I) catalyzed Huisgen 1,3-dipolar cycloaddition under classical Sharpless conditions, yielding respectively high amounts of triazole linked bithiophenes **4a**–**c** and **5a**,**b** (Scheme 1) [44].

### 2.1. Formation of Free Thiols and Disulfides

The self organization of SAMs on gold surfaces essentially requires the presence of free thiols or disulfides [45,46,47,48,49,50]. Consequently thioacetate derivatives **4a–c** as well as **5a**,**b** needed to be transformed into their corresponding thiols **2a–c** and **3a**,**b** or disulfides **11a–c** and **12a**,**b**. This was facilitated by mild alkaline hydrolysis as outlined in Scheme 1. Whether free thiols or disulfides were obtained primarily depended on the duration of the reaction. Short reaction times (≤2 min) selectively promoted the formation of thiols, while extended reaction times (≥90 min) led to gradual oxidation of these thiols and the formation of the corresponding disulfides.

### 2.2. Monolayer Formation and Properties

The bithiophene chromophore represents a core structure of the synthesized molecules and has been selected due to its specific absorption maxima. Precursors **4a–c** and **5a**,**b** and their follow up products displayed an absorption maximum of 340 nm (ξmax ≈ 21,000 L·mol^−1^·cm^−1^) [3,51], which is close to the emission wavelength of a standard nitrogen laser (337 nm). Additionally the bithiophene moiety exhibited useful electrochemical properties that permitted the use of cyclic voltammetry (CV) for studying the redox behavior of any synthesized compounds. It further allowed evaluation of the surface coverage and long term stability of immobilized **2a–c** and **3a**,**b**. As expected, CV of triazolobithiophenes **4a–c** and **5a**,**b** in solution exhibited a reversible one-electron oxidation wave around 0.49 V (*vs*. Fc+/Fc) [3]. This characteristic one electron-transfer process confirmed the redox properties of the bithiophenic chromophore.

**Figure 2:**
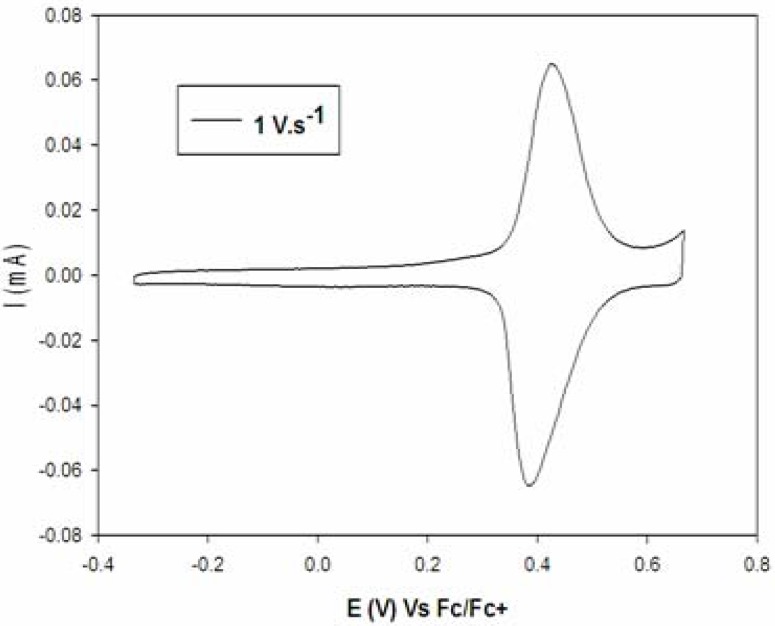
Cyclic voltammogram of immobilized **12b** (5 × 10^−4^ M in CH_2_Cl_2_).

The formation of SAMs was then performed using freshly prepared gold electrodes, which were incubated for 30 min at 20 °C in a CH_2_Cl_2_ solution (5·10^−4^ M) of thiols **2a–c**, **3a**,**b** or disulfides **11a–c** and **12a**,**b**. Figure 2 displays the cyclic voltammogram of immobilized **12b**, showing a defined first reversible oxidation wave, which is characteristic for a surface confined redox system. In addition, the anodic-cathodic peaks separation was close to 0 volt, confirming that the redox process of the bithiophenic system was not limited by charge transport. Further, high surface coverage (Γ) (Table 1) was observed (about 3.8 × 10^−10^ mol·cm^−2^) indicating a good organization of SAMs [49]. Monolayers were stable for at least 48 h regardless whether they originated from disulfides (**11b**,**c**, **12a**,**b**) or their corresponding thiols **2b–c**, **3a**,**b**. Only thiol **2a** and its corresponding disulfide **11a** did not produce stable monolayers. Furthermore, the stability of the triazolobithiophenic SAMs is enhanced by a spacer of at least two carbons between the triazole ring and the bithiophene moiety. This may allow a better packing arrangement of SAMs on the gold surface. In accordance to that, **2a** and **11a** did not produce stable monolayers.

**Table 1:** Redox potentials (**E_1_^ox^**) ^a^ and covering rate (**Γ**) ^a^ for SAMs of immobilized **11a–c** and **12a**,**b**.

Compounds	E_1_^ox^	Γ.10^−10^
**11a**	0.52	Not stable
**11b**	0.45	3.8 ± 0.1
**11c**	0.41	3.6 ± 0.1
**12a**	0.50	3.8 ± 0.3
**12b**	0.41	3.8 ± 0.2

*^a^* The data of potentials (**E_1_^ox^**) are displayed in volt versus Fc^+^/Fc in CH_2_Cl_2_; using Bu_4_NPF_6_ (0.1 M) as the supporting electrolyte on gold working electrode; scan rate 500 mV/s. The covering rate (**Γ**) was calculated in mol·cm^−2^.

### 2.3. MS Application

Compounds **2a–c** and **3a**,**b** have been specifically designed to study the principle of energy transmission in light absorbing monolayers. In this context, DIAMS has been described as a new laser desorption MS method [2,3,49]. Desorption/ionization on self-assembled monolayer surfaces aims for the elimination of matrix noise present in classical MALDI spectra, by replacing matrix molecules with light absorbing SAMs. Hithero, **1** represents the only light absorbing molecule reported for that purpose [2,3]. Benefitting from their improved physical properties, **2b**,**c** and **3a**,**b** as well as **11b**,**c** and **12a**,**b** are currently employed for thorough MS studies on their light absorption and energy transmission principle. Figure 2 shows the time of flight mass spectrum of the alkaloid yohimbine (C_21_H_26_N_2_O_3_). The compound was directly deposited onto freshly immobilized **3b** and ionized by a laser beam at 337 nm, a wavelength at which yohimbine does not absorb light [50]. Only the pseudo-molecular ion [M−H]^+^ at *m/z* 353.21, but no fragmentation products or signals from the monolayer were detected. The stability of the SAMs was also confirmed by recording a spectrum without any analyte at the same laser attenuation (70%). As previously observed for bithiophenic SAM **1** [2], at this level of laser energy, ions characteristic of the monolayer degradation are not detected.

**Figure 3:**
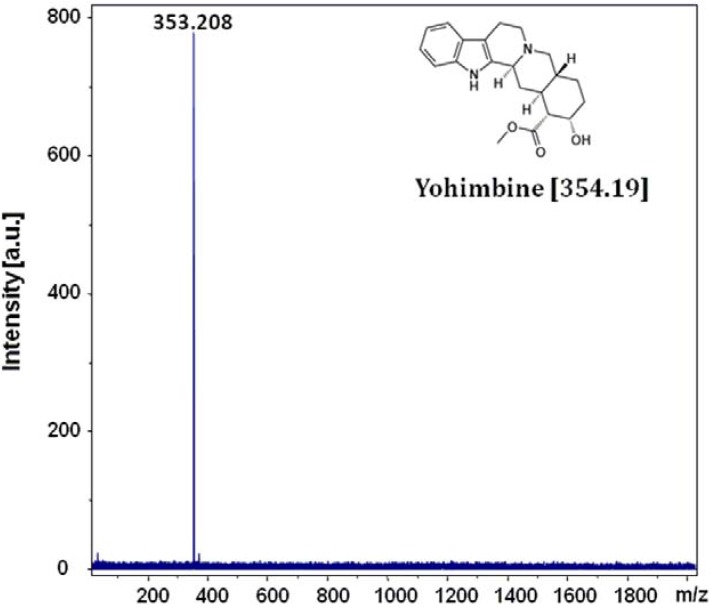
Mass spectrum of yohimbine acquired by laser desorption ionization from immobilized **3b** in linear positive mode (C_21_H_26_N_2_O_3_ calculated isotopic mass: 354.19, observed: [M−H]^+^: 353.21. Laser energy 30% (46.8 µJ)). On first sight the pseudomolecular ion [M−H]^+^ appears unusual, nevertheless its occurrence is essentially linked to the process of ion formation in laser desorption MS with photochemical interactions between matrix and analyte molecules [54].

## 3. Experimental

### 3.1. General

All reagents and solvents were of commercial quality. DMF and MeOH were dried on molecular sieves (4Å). Column chromatography (CC) was performed on silica gel 60 (Merck, particle diameter 0.035–0.070 mm). Analytical TLC was carried out on standard silica gel 60 plates (Merck silica gel 60 F254) and compounds were visualized under UV light (254/366 nm). In addition plates were sprayed with an ethanolic solution of sulfuric acid (10%) and heated for 1 min at 100 °C. Melting points were determined using a WME-type Köffler hot stage (Wagner&Munz). ^1^H- and ^13^C-NMR spectra were obtained at room temperature on a Bruker Avance III 300 or a Bruker Avance DRX 500 spectrometer. Infrared spectra were recorded in the range of 4,000–400 cm^−1^ using potassium bromide disks or ATR, with a Bruker FT IR Vector 22 spectrophotometer. UV-Vis absorption was recorded on a Perkin-Elmer Lambda 19 and on a Perkin-Elmer Lambda 950 spectrometer. Exact mass measurements were performed on a LTQ-Orbitrap MS (Thermo Fisher Scientific) or on a Bruker MicrOTOF-Q II MS. The DIAMS mass spectrum of the alkaloid yohimbine was recorded on a Bruker Biflex III time-of-flight mass spectrometer (Bruker-Daltonics) equipped with a nitrogen laser (337 nm, model VSL-337i, Laser Science Inc.).

### 3.2. Electrochemistry

Electrochemical experiments were carried out with a Biologic SP-150 potentiostat at 20 °C. Cyclic voltammetry was performed in a three-electrode cell equipped with a platinum-plate counter electrode and an Ag/AgNO_3_ (0.01 M CH_3_CN) reference electrodes. Cyclic voltammograms were recorded in HPLC-grade methylene chloride (CH_2_Cl_2_) using Bu_4_NPF_6_ as supporting electrolyte. Gold electrodes were prepared by physical vapor deposition as previously described. In short, borosilicate glass plates were covered with a chrome layer of about 5 nm, followed by a gold layer of approximately 50 nm. Fresh electrodes were prepared just before the experiments.

### 3.3. Synthesis

#### 3.3.1. Alkynylbithiophenes **6a–c**

3-(5'-(Methylthio)-2,2'-bithiophen-5-ylthio)propionitrile (**8**, 150 mg, 0.51 mmol, 1 equiv.) was dissolved in dry DMF (13 mL) and the solution was sparged with nitrogen for 45 min. Then an oxygen free methanolic solution (7 mL) of cesium hydroxide (128 mg, 0.76 mmol, 1.5 equiv.) was added and the mixture was stirred at room temperature for 1 h. Finally, 3-bromoprop-1-yne (121 mg, 1.02 mmol, 2 equiv.) was added. The solution was chilled to a temperature of −40 °C and stirred for another hour. The mixture was diluted with water (40 mL) and extracted with AcOEt (3 × 40 mL). The combined organic phases were washed with water (3 × 50 mL) and dried over MgSO_4_. After solvent removal, the residue was purified by isocratic CC (petroleum ether/CH_2_Cl_2_: 80/20) yielding *5-(methylthio)-5'-(prop-2-ynylthio)-2,2'-bithiophene* compound (**6a**). Yield 86 %; green brownish oil; C_12_H_10_S_4_; IR (ATR): 791, 869, 964, 1070, 1196, 1225, 1313, 1420, 1497, 2918, 3066, 3288 cm^−1^; ^1^H-NMR (CDCl_3_/300 MHz): δ 2.31 (t, *J* = 2.7 Hz, 1H), 2.51 (s, 3H), 3.49 (d, *J* = 2.4 Hz, 2H), 6.96–7.17 (m, 4H); ^13^C-NMR (CDCl_3_/75 MHz): δ 21.9, 27.4, 72.7, 79.5, 123.8, 124.2, 131.3, 131.5, 136.1, 137.2, 138.7, 141.8. MALDI-TOF-MS positive mode [C_12_H_10_S_4_]^+^: 281.9 *m/z*.

Compounds **6b** and **6c** were prepared following procedure of **6a** with slight modifications. For compound **6b**, 4-bromobut-1-yne was added to the cesium hydroxide solution at 0 °C. The mixture was stirred overnight at room temperature. Compound **6c** was obtained under the same conditions using 4 equiv. of 5-chloropent-1-yne. The mixture was stirred at room temperature for 24 h.

*5-(But-3-ynylthio)-5'-(methylthio)-2,2'-bithiophene* (**6b**). Yield 94%; green yellowish solid; C_13_H_12_S_4_; mp: 68–70 °C; IR (ATR): 791, 865, 966, 1196, 1220, 1275, 1312, 1422, 1497, 1688, 2853, 2921, 3278 cm^-1^; ^1^H-NMR (CDCl_3_/500 MHz): δ 2.04 (t, *J* = 2.5 Hz, 1H), 2.50 (dt, *J* = 7.0 and 2.5 Hz, 2H), 2.51 (s, 3H), 2.92 (t, *J* = 7.0 Hz, 2H), 6.96–7.06 (m, 4H); ^13^C-NMR (CDCl_3_/125 MHz): δ 19.4, 22.0, 37.2, 69.7, 81.9, 123.7, 124.0, 131.6, 132.1, 135.5, 137.0, 138.7, 141.1. MALDI-TOF-MS positive mode [C_13_H_12_S_4_]^+^: 295.9 *m/z*.

*5-(Methylthio)-5'-(pent-4-ynylthio)-2,2'-bithiophene* (**6c**). Yield 85%; green brownish oil; C_14_H_14_S_4_; IR (ATR): 792, 868, 964, 1196, 1212, 1343, 1423, 1497, 2852, 2918, 3067, 3294 cm^−1^; ^1^H-NMR (CDCl_3_/500 MHz): δ 1.85 (qu, *J* = 7.0 Hz, 2H), 1.97 (t, *J* = 2.5Hz, 1H), 2.35 (dt, *J* = 7.0 and 2.5 Hz, 2H), 2.51 (s, 3H), 2.91 (t, *J* = 7 Hz, 2H), 6.96–7.01 (m, 4H); ^13^C-NMR (CDCl_3_/125 MHz): δ 17.1, 22.0, 27.9, 37.5, 69.1, 83.1, 123.7, 123.9, 131.6, 133.4, 134.5, 136.9, 138.9, 140.4. MALDI-TOF-MS positive mode [C_14_H_14_S_4_]^+^: 310.0 *m/z*.

#### 3.3.2. Azidobithiophenes **7a–b**

Azidobithiophenes **7a** and **7b** were prepared with slight modifications according to the general procedure described for alkynylbithiophenes **6a–c**. Alternatively, 2-azidoethyl-4-methylbenzenesulfonate (for **7a**) or 3-azidopropyl-4-methylbenzenesulfonate (for **7b**) was added to the cesium hyroxyde solution of **8**. The mixtures were kept at room temperature and stirred for 4 h (**7a**) or 24 h (**7b**). Finally, compounds **7a** and **7b** were purified by isocratic CC (cyclohexane/CH_2_Cl_2_: 90/10).

*5-(2-Azidoethylthio)-5'-(methylthio)-2,2'-bithiophene* (**7a**). Yield 99%; green solid; C_11_H_11_N_3_S_4_; mp: 44–46 °C; IR (ATR): 794, 868, 964, 1224, 1256, 1423, 1498, 1687, 2099, 2855, 2921, 3067 cm^−1^; ^1^H-NMR (CDCl_3_/270 MHz): δ 2.50 (s, 3H), 2.94 (t, *J* = 6.9 Hz, 2H), 3.47 (t, *J* = 6.9 Hz, 2H), 6.97–7.26 (m, 4H); ^13^C-NMR (CDCl_3_/67.5 MHz): δ 21.9, 37.7, 50.0, 123.8, 124.2, 131.0, 131.5, 135.8, 137.2, 138.5, 141.4. MALDI-TOF-MS positive mode [C_11_H_11_N_3_S_4_]^+^: 312.9 *m/z*.

*5-(3-Azidopropylthio)-5'-(methylthio)-2,2'-bithiophene* (**7b**). Yield 76%; green oil; C_12_H_13_N_3_S_4_; IR (ATR): 792, 867, 964, 1069, 1245, 1421, 1498, 1687, 2092, 2859, 2920, 3068 cm^−1^; ^1^H-NMR (CDCl_3_/270 MHz): δ 1.89 (qu, *J* = 7.0 Hz, 2H), 2.50 (s, 3H), 2.87 (t, *J* = 7.0 Hz, 2H), 3.44 (t, *J* = 7.0 Hz, 2H), 6.97–7.01 (m, 4H); ^13^C-NMR (CDCl_3_/67.5 MHz): δ 21.9, 28.4, 35.6, 49.5, 123.7, 123.9, 131.5, 132.7, 134.8, 136.9, 138.9, 140.6. MALDI-TOF-MS positive mode [C_12_H_13_N_3_S_4_]^+^: 327.0 *m/z*.

#### 3.3.3. 8-Azidooctyl Thioacetate (**9**)

8-Bromooctyl thioacetate (2.75 g, 10.3 mmol, 1 equiv.) was dissolved in dry DMF (100 mL). After the addition of sodium azide (1 g, 15.3 mmol, 1.5 equiv.), the mixture was heated to 110 °C overnight. After cooling to room temperature, distilled water (100 mL) was added. The mixture was extracted with diethyl ether (2 × 150 mL), and the combine organic layers were washed with distilled water (2 × 150 mL) and dried over MgSO_4_. After solvent evaporation, the residue was purified by isocratic CC (AcOEt/petroleum ether: 5/95). Yield 89%; yellowish liquid; C_10_H_19_N_3_OS; IR (ATR): 954, 1133, 1260, 1352, 1460, 1690, 2092, 2856, 2930 cm^−1^; ^1^H-NMR (CDCl_3_/500 MHz): δ 1.19–1.37 (m, 8H), 1.53–1.62 (m, 4H), 2.32 (s, 3H), 2.86 (t, *J* = 7.0 Hz, 2H), 3.25 (t, *J* = 7.0 Hz, 2H); ^13^C-NMR (CDCl_3_/125 MHz): δ 26.6, 28.6, 28.7, 28.9, 28.9, 29.0, 29.4, 30.6, 51.4, 195.9. HRMS (ESI^+^): calcd for [C_10_H_19_N_3_OS+Na]^+^: 252.1141, found 252.1143.

#### 3.3.4. Dec-9-ynyl Thioacetate (**10**)

Dec-9-ynyl methanesulfonate (0.2 g, 0.86 mmol, 1 equiv.) was dissolved in dry DMF (10 mL). After the addition of 20 mL oxygen free potassium thioacetate solution (1.1 equiv., 0.95 mmol) in dry DMF, the mixture underwent microwave irradiation (400 W, 100 °C, 30 min). After solvent evaporation, the residue was purified by isocratic CC (cyclohexane/AcOEt: 90/10). Yield 50%; orange brownish liquid; C_12_H_20_OS; IR (ATR): 954, 1133, 1353, 1433, 1460, 1690, 2856, 2929, 3301 cm^−1^; ^1^H-NMR (CDCl_3_/270 MHz): δ 1.20–1.37 (m, 8H), 1.42–1.53 (m, 4H), 1.89 (t, *J* = 2.7 Hz, 1H), 2.09–2.15 (m, 2H), 2.27 (s, 3H), 2.81 (t, *J* = 7.3 Hz, 2H); ^13^C-NMR (CDCl_3_/67.5 MHz): δ 17.8, 27.8, 28.0, 28.1, 28.3, 28.4, 28.5, 28.9, 30.0, 67.6, 84.0, 195.3. MALDI-TOF-MS negative mode [C_12_H_20_OS-H]^−^: 211.0 *m/z*.

#### 3.3.5. Triazolobithiophenes **4a–c**

An aqueous solution (2.5 mL) containing CuSO_4_.5H_2_O (12 mg, 0.05 mmol, 0.12 equiv.) and sodium ascorbate (29 mg, 0.15 mmol, 0.36 equiv.) were added to a mixture of alkynylbithiophene **6a** (125 mg, 0.44 mmol, 1 equiv.) and azidothioacetate **9** (107 mg, 0.47 mmol, 1.06 equiv.) in *t*BuOH/H2O (4 mL/1.5 mL). The mixture was stirred overnight at room temperature yielding a green brownish solid. The latter was first filtered and then washed with distilled water (4 × 25 mL) and diethyl ether (10 mL). After being vacuum dried at 50 °C for several hours, the residue was identified as triazolobithiophene **4a**. Triazolobithiophenes **4b** and **4c** were obtained following the same procedure, but starting respectively from alkynylbithiophenes **6b** and **6c**.

*S-8-(4-((5'-(Methylthio)-2,2'-bithiophen-5-ylthio)methyl)-1H-1,2,3-triazol-1-yl)octyl thioacetate* (**4a**). Yield 83%; pale green brownish solid; C_22_H_29_ON_3_S_5_; mp: 85–87 °C; UV-Vis (CH_2_Cl_2_): 345 nm; IR (ATR): 791, 867, 961, 1056, 1142, 1352, 1422, 1464, 1691, 2851, 2920, 3069, 3124 cm^−1^; ^1^H-NMR (CDCl_3_/500 MHz): δ 1.28 (s, 8H), 1.53–1.59 (m, 2H), 1.81–187(m, 2H), 2.32 (s, 3H), 2.51 (s, 3H), 2.85 (t, *J* = 7.0 Hz, 2H), 4.10 (s, 2H), 4.29 (t, *J* = 7.0 Hz, 2H), 6.92–6.95 (m, 4H), 7.28(s, 1H); ^13^C-NMR (CDCl_3_/125 MHz): δ 21.9, 26.3, 28.6, 28.7, 28.8, 29.0, 29.4, 30.3, 30.6, 33.7, 50.3, 121.9, 123.6, 124.1, 131.5, 132.3, 135.4, 137.1, 138.7, 141.1, 196.0. HRMS (ESI^+^): calcd for [C_22_H_29_ON_3_S_5_+H]^+^: 512.0987, found 512.0984.

*S-8-(4-(2-(5'-(Methylthio)-2,2'-bithiophen-5-ylthio)ethyl)-1H-1,2,3-triazol-1-yl)octyl thioacetate* (**4b**). Yield 95%; pale green brownish solid; C_23_H_31_ON_3_S_5_; mp: 85–87 °C; UV-Vis (CH_2_Cl_2_): 344 nm; IR (ATR): 816, 864, 960, 1055, 1143, 1213, 1269, 1426, 1464, 1694, 2359, 2851, 2922, 3148 cm^−1^; ^1^H-NMR (CDCl_3_/500 MHz): δ 1.30 (s, 8H), 1.54 (qu, *J* = 7.0 Hz, 2H), 1.86–1.89 (m, 2H), 2.31 (s, 3H), 2.51 (s, 3H), 2.84 (t, *J* = 7.0 Hz, 2H), 3.05 (t, *J* = 7.0 Hz, 2H), 3.12 (t, *J* = 7.0 Hz, 2H), 4.31(t, *J* = 7.0 Hz, 2H), 6.96–7.03 (m, 4H), 7.36(s, 1H); ^13^C-NMR (CDCl_3_/125 MHz): δ 22.0, 25.9, 26.4, 28.6, 28.8, 28.9, 29.0, 29.4, 30.2, 30.6, 38.1, 50.2, 121.2, 123.8, 123.9, 131.6, 132.9, 134.8, 136.9,138.8, 140.6, 145.5, 196.0. HRMS (ESI^+^): calcd for [C_23_H_31_ON_3_S_5_+H]^+^: 526.1143, found 526.1145.

*S-8-(4-(3-(5'-(Methylthio)-2,2'-bithiophen-5-ylthio)propyl)-1H-1,2,3-triazol-1-yl)octyl thioacetate* (**4c**). Yield 70%; pale green solid; C_24_H_33_ON_3_S5; mp: 84–86 °C; UV-Vis (CH_2_Cl_2_): 344 nm; IR (ATR): 792, 862, 960, 1059, 1143, 1207, 1351, 1425, 1461, 1691, 2850, 2921, 3069, 3120 cm^−1^; ^1^H-NMR (CDCl3/500 MHz): δ 1.29 (s, 8H), 1.52–1.58 (m, 2H), 1.84–1.88 (m, 2H), 2.03 (qu, *J* = 7.0 Hz, 2H), 2.32 (s, 3H), 2.51 (s, 3H), 2.83-2.87 (m, 6H), 4.29 (t, *J* = 7.0 Hz, 2H), 6.96–7.01 (m, 4H); 7.25 (s, 1H); ^13^C-NMR (CDCl_3_/125 MHz): 22.0, 24.1, 26.4, 28.6, 28.7, 28.8, 28.9, 29.0, 29.39, 30.2, 30.6, 38.0, 50.2, 120.7, 123.7, 123.9, 131.6, 133.6, 134.4, 136.8, 138.8, 140.2, 195.9. HRMS (ESI^+^): calcd for [C_24_H_33_ON_3_S_5_+H]^+^: 540.1299, found 540.1296.

#### 3.3.6. Triazolobithiophenes **5a–b**

Triazolobithiophenes **5a** and **5b** were prepared following the procedure previously described for triazolobithiophenes **4a–c**, but using azidobithiophenes **7a**,**b** and dec-9-ynyl thioacetate **10** instead of **6a–c** and **9**. Moreover, the reaction time was extended to 36 h.

*S-8-(1-(2-(5'-(Methylthio)-2,2'-bithiophen-5-ylthio)ethyl)-1H-1,2,3-triazol-4-yl)octyl thioacetate* (**5a**). Yield 91%; pale green brownish solid; C_23_H_31_ON_3_S_5_; mp: 80–82 °C; UV-Vis (CH_2_Cl_2_): 343 nm; IR (KBr): 785, 866, 956, 1053, 1136, 1218, 1353, 1689, 2849, 2923, 3062, 3114 cm^−1^; ^1^H-NMR (CDCl_3_/270 MHz): δ 1.30 (s, 8H), 1.54 (t, *J* = 6.8 Hz 2H), 1.65 (m, 2H), 2.31 (s, 3H), 2.51 (s, 3H), 2.70 (t, *J* = 7.6 Hz, 2H), 2.84 (t, *J* = 7.2 Hz, 2H), 3.24 (t, *J* = 6.6 Hz, 2H), 4.53 (t, *J* = 6.6 Hz, 2H), 6.97–7.06 (m, 4H), 7.33 (s, 1H); ^13^C-NMR (CDCl_3_/67.5 MHz): δ 21.9, 25.6, 28.7, 28.9, 29.1, 29.3, 29.4, 30.6, 38.2, 49.0, 64.0, 71.4, 121.2, 123.8, 124.3, 130.8, 131.4, 135.8, 137.4, 138.2, 141.5, 148.3, 196.0. HRMS (ESI^+^): calcd for [C_23_H_31_ON_3_S_5_+H]^+^: 526.1143, found 526.1141.

*S-8-(1-(3-(5'-(Methylthio)-2,2'-bithiophen-5-ylthio)propyl)-1H-1,2,3-triazol-4-yl)octyl thioacetate* (**5b**). Yield 81%; pale green brownish solid; C_24_H_33_ON_3_S_5_; mp: 80–82 °C; UV-Vis (CH_2_Cl_2_): 343 nm; IR (KBr): 785, 865, 955, 1052, 1125, 1212, 1354, 1467, 1689, 2850, 2920, 3064, 3116 cm^−1^; ^1^H-NMR (CDCl_3_/270 MHz): δ 1.31 (s, 8H), 1.55 (t, *J* = 4.0Hz, 2H), 1.65 (m, 4H), 2.24 (m, 2H), 2.32 (s, 3H), 2.51 (s, 3H), 2.75–2.82 (m, 2H), 2.86 (t, *J* = 3.6 Hz, 2H), 4.51 (s, 2H), 6.97–7.03 (m, 4H), 7.33 (s, 1H); ^13^C-NMR (CDCl_3_/67.5 MHz): δ 21.9, 25.6, 28.7, 28.9, 29.0, 29.1, 29.3, 29.4, 29.5, 30.6, 35.3, 38.3, 48.1, 64.0, 120.9, 123.8, 124.1, 131.5, 132.3, 135.0, 137.2, 138.6, 140.9, 195.9. HRMS (ESI^+^): calcd for [C_24_H_33_ON_3_S_5_+H]^+^: 540.1299, found 540.1295.

#### 3.3.7. Thiols **2a–c** and **3a,b**

Triazole **4c** (20 mg, 37 mmol, 1 equiv.) was dissolved in dry DMF (2 mL) ventilated with nitrogen for 45 min. Then, 1 mL of a methanolic cesium hydroxide solution (22 mg, 0.13 mmol, 3.5 equiv.) was added. The mixture was stirred for 2 min at room temperature. The reaction was quenched by addition of 0.2% HCl solution (5 mL). The solid was filtered and washed with distilled water (5 mL). In a second purification step, the residue was solubilized in CH_2_Cl_2_ (20 mL), and washed with distilled water (4 × 20 mL). Finally the organic phase was dried using MgSO_4_. After solvent removal, **2c** was obtained.

Following the same procedure, the hydrolysis of **4a**,**b** and **5a**,**b** yielded respectively **2a**,**b** and **3a**,**b**.

*8-(4-((5'-(Methylthio)-2,2'-bithiophen-5-ylthio)methyl)-1H-1,2,3-triazol-1-yl)octane-1-thiol* (**2a**). Yield 55%; pale yellow solid; C_20_H_27_N_3_S_5_; mp: 80–82 °C; IR (ATR): 789, 863, 1055, 1141, 1227, 1425, 1459, 1600, 2853, 2919, 3073, 3124 cm^−1^; ^1^H-NMR (CDCl_3_/300 MHz): δ 1.25–1.37 (m, 8H), 1.53–1.63 (m, 2H), 1.81–188 (m, 2H), 2.50 (s, 3H), 2.51 (pseudo q, *J* = 7.5 Hz, 2H), 4.10 (s, 2H), 4.29 (t, *J* = 7.2 Hz, 2H), 6.90–6.95 (m, 4H), 7.26 (s, 1H); ^13^C-NMR (CDCl_3_/75 MHz): δ 21.9, 26.3, 28.3, 28.8, 28.9, 29.1, 30.3, 33.7, 38.9, 50.3, 121.9, 123.6, 124.1, 131.5, 132.2, 135.4, 137.1, 138.6, 141.1, 144.1. HRMS (ESI^−^): calcd for [C_20_H_27_N_3_S_5_-H]^−^: 468.0736, found 468.0736.

*8-(4-(2-(5'-(Methylthio)-2,2'-bithiophen-5-ylthio)ethyl)-1H-1,2,3-triazol-1-yl)octane-1-thiol* (**2b**). Yield 44%; brown yellowish solid; C_21_H_29_N_3_S_5_; mp: 79–81 °C; IR (ATR): 796, 865, 1035, 1099, 1211, 1257, 1314, 1427, 1632, 2850, 2919, 3071, 3143 cm^−1^; ^1^H-NMR (CDCl_3_/300 MHz): δ 1.25–1.34 (m, 8H), 1.54–1.64 (m, 2H), 1.85–1.90 (m, 2H), 2.51 (pseudo q, *J* = 7.5 Hz, 2H), 2.51 (s, 3H), 3.01–3.15 (m, 4H), 4.31 (t, *J* = 7.2 Hz, 2H), 6.95–7.03 (m, 4H), 7.35 (s, 1H); ^13^C-NMR (CDCl_3_/75 MHz): δ 22.0, 25.9, 26.4, 28.3, 28.8, 28.9, 29.0, 30.3, 38.1, 38.9, 50.2, 121.2, 123.8, 123.9, 131.6, 132.9, 134.8, 136.9, 138.8, 140.6, 145.5. HRMS (ESI^-^): calcd for [C_21_H_29_N_3_S_5_-H]^−^: 482.0892, found 482.0892.

*8-(4-(3-(5'-(Methylthio)-2,2'-bithiophen-5-ylthio)propyl)-1H-1,2,3-triazol-1-yl)octane-1-thiol* (**2c**). Yield 41%; brown yellowish solid; C_22_H_31_N_3_S_5_; mp: 76–78 °C; IR (KBr): 792, 861, 993, 1059, 1157, 1205, 1424, 1459, 1552, 2849, 2921, 3067, 3119 cm^−1^; ^1^H-NMR (CDCl_3_/300 MHz): δ 1.24–1.35 (m, 8H), 1.54-1.63 (m, 2H), 1.84–1.88 (m, 2H), 2.02 (qu, *J* = 7.2 Hz, 2H), 2.50 (s, 3H), 2.51 (pseudo q, *J* = 7.5 Hz, 2H), 2.82-2.87 (m, 4H), 4.28 (t, *J* = 7.2 Hz, 2H), 6.96–7.01 (m, 4H), 7.23 (s, 1H); ^13^C-NMR (CDCl_3_/75 MHz): δ 22.0, 24.1, 26.4, 28.3, 28.8, 28.9, 29.0, 29.1, 30.3, 38.0, 38.9, 50.2, 120.7, 123.7, 123.9, 131.6, 133.7, 134.4, 136.8, 138.9, 140.2, 146.8. HRMS (ESI^−^): calcd for [C_22_H_31_N_3_S_5_-H]^−^: 496.1049, found 496.1048.

*8-(1-(2-(5'-(Methylthio)-2,2'-bithiophen-5-ylthio)ethyl)-1H-1,2,3-triazol-4-yl)octane-1-thiol* (**3a**). Yield 44%; brownish solid; C_21_H_29_N_3_S_5_; mp: 76–78 °C; IR (ATR): 790, 865, 988, 1058, 1105, 1221, 1316, 1426, 1550, 2852, 2922, 3066, 3113 cm^−1^; ^1^H-NMR (CDCl_3_/300 MHz): δ 1.24–1.34 (m, 8H), 1.57–1.68 (m, 4H), 2.51 (pseudo q, *J* = 7.5 Hz, 2H), 2.51 (s, 3H), 2.64–2.72 (m, 2H), 3.24 (t, *J* = 6.9 Hz, 2H), 4.53 (t, *J* = 6.9 Hz, 2H), 6.96–7.07 (m, 4H), 7.32 (s, 1H); ^13^C-NMR (CDCl_3_/75 MHz): 21.9, 25.6, 28.4, 29.1, 29.3, 29.4, 29.7, 32.7, 38.3, 39.1, 49.1, 121.2, 123.9, 124.3, 130.8, 131.5, 135.9, 137.5, 138.3, 141.5, 148.3. HRMS (ESI^-^): calcd for [C_21_H_29_N_3_S_5_-H]^−^: 482.0892 , found 482.0892.

*8-(1-(3-(5'-(Methylthio)-2,2'-bithiophen-5-ylthio)propyl)-1H-1,2,3-triazol-4-yl)octane-1-thiol* (**3b**). Yield 43%; pale yellow solid; C_22_H_31_N_3_S_5_; mp: 74–76 °C; IR (ATR): 792, 860, 907, 1037, 1170, 1211, 1425, 1495, 1548, 2850, 2921, 3066, 3116 cm^−1^; ^1^H-NMR (CDCl_3_/300 MHz): δ 1.30–1.35 (m, 8H), 1.57–1.64 (m, 4H), 2.17–2.24 (m, 2H), 2.51 (s, 3H), 2.51 (pseudo q, *J* = 7.5 Hz, 2H), 2.64–2.71 (m, 2H), 2.76 (t, *J* = 6.9 Hz, 2H), 4.46 (t, *J* = 6.9 Hz, 2H), 6.97–7.03 (m, 4H), 7.23 (s, 1H); ^13^C-NMR (CDCl_3_/75 MHz): δ 18.5, 22.1, 25.7, 28.5, 29.2, 29.3, 29.5, 29.6, 35.4, 39.2, 48.2, 58.6, 120.9, 123.9, 124.2, 131.6, 132.3, 135.2, 137.3, 138.6, 141.0, 148.6. HRMS (ESI^-^): calcd for [C_22_H_31_N_3_S_5_-H]^−^: 496.1049, found 496.1050.

#### 3.3.8. Disulfides **11a–c, 12a,b**

The reaction was carried out described for thiols **2a–c**, and **3a**,**b** under slightly modified conditions. The reaction time was increased to 90 min and 3 equiv. cesium hydroxide (19 mg, 0.11 mmol) were used. These conditions finally yielded disulfides **11a–c** and **12a**,**b**, respectively.

*1,2-Bis(8-(4-((5'-(methylthio)-2,2'-bithiophen-5-ylthio)methyl)-1H-1,2,3-triazol-1-yl)octyl)disulfide* (**11a**). Yield 44%; pale yellow solid; C_40_H_52_N_6_S_10_; mp: 122–124 °C; IR (KBr): 791, 867, 1057, 1226, 1312, 1342, 1423, 1462, 1497, 1549, 2849, 2918, 3069, 3122 cm^−1^; ^1^H-NMR (CDCl_3_/300 MHz): δ 1.29 (s, 8H), 1.58–1.65 (m, 2H), 1.81–186 (m, 2H), 2.50 (s, 3H), 2.66 (t, *J* = 6.9 Hz, 2H), 4.10 (s, 2H), 4.29 (t, *J* = 6.9 Hz, 2H), 6.92–6.95 (m, 4H), 7.26 (s, 1H); ^13^C-NMR (CDCl_3_/75 MHz): δ 21.9, 26.3, 28.3, 28.8, 28.9, 29.1, 30.3, 33.7, 38.9, 50.3, 121.9, 123.6, 124.1, 131.5, 132.2, 135.4, 137.1, 138.6, 141.1, 144.1. HRMS (ESI^+^): calcd for [C_40_H_52_N_6_S_10_+H]^+^: 937.1540, found 937.1544.

*1,2-Bis(8-(4-(2-(5'-(methylthio)-2,2'-bithiophen-5-ylthio)ethyl)-1H-1,2,3-triazol-1-yl)octyl)disulfide* (**11b**). Yield 50%; brown yellowish solid; C_42_H_56_N_6_S_10_; mp: 118–120 °C; IR (KBr): 792, 865, 1055, 1144, 1214, 1268, 1316, 1427, 1498, 1551, 2850, 2920, 3067, 3146 cm^−1^; ^1^H-NMR (CDCl_3_/500 MHz): δ 1.25–1.36 (m, 8H), 1.57–1.67 (m, 2H), 1.87–1.90 (m, 2H), 2.51 (s, 3H), 2.65 (t, *J* = 7.5 Hz, 2H), 3.06 (t, *J* = 7 Hz, 2H), 3.13 (t, *J* = 6.5 Hz, 2H), 4.32 (t, *J* = 7.5 Hz, 2H), 6.96–7.03 (m, 4H), 7.38 (s, 1H); ^13^C-NMR (CDCl_3_/125 MHz): δ 22.0, 25.9, 26.4, 28.3, 28.8, 28.9, 29.0, 30.3, 38.1, 38.9, 50.2, 121.2, 123.8, 123.9, 131.6, 132.9, 134.8, 136.9, 138.8, 140.6, 145.5 ppm. HRMS (ESI^+^) calcd for [C_42_H_56_N_6_S_10_+H]^+^: 965.1846, found 965.1855.

*1,2-Bis(8-(4-(3-(5'-(methylthio)-2,2'-bithiophen-5-ylthio)propyl)-1H-1,2,3-triazol-1-yl)octyl)disulfide* (**11c**). Yield 40%; brown yellowish solid; C_44_H_60_N_6_S_10_; mp: 118–120 °C; IR (KBr): 790, 865, 1026, 1059, 1150, 1211, 1424, 1460, 1497, 1550, 2849, 2920, 3068, 3123 cm^−1^; ^1^H-NMR (CDCl_3_/300 MHz): δ 1.31 (s, 8H), 1.58–1.68 (m, 2H), 1.84–1.89 (m, 2H), 2.02 (qu, *J* = 7.2 Hz, 2H), 2.50 (s, 3H), 2.66 (t, *J* = 7.5 Hz, 2H), 2.82–2.88 (m, 4H), 4.28 (t, *J* = 7.2 Hz, 2H), 6.96–7.01 (m, 4H), 7.23 (s, 1H); ^13^C-NMR (CDCl_3_/75 MHz): δ 22.0, 24.1, 26.4, 28.3, 28.8, 28.9, 29.0, 29.1, 30.3, 38.0, 38.9, 50.2, 120.7, 123.7, 123.9, 131.6, 133.7, 134.4, 136.8, 138.9, 140.2, 146.8. HRMS (ESI^+^) calcd for [C_44_H_60_N_6_S_10_+H]^+^: 993.2160, found 993.2161.

*1,2-Bis(8-(1-(2-(5'-(methylthio)-2,2'-bithiophen-5-ylthio)ethyl)-1H-1,2,3-triazol-4-yl)octyl)disulfide* (**12a**). Yield 66%; brown yellowish solid; C_42_H_56_N_6_S_10_; mp: 125–127 °C; IR (KBr): 794, 865, 1052, 1096, 1212, 1261, 1423, 1464, 1496, 1548, 2850, 2921, 3062, 3119 cm^−1^; ^1^H-NMR (CDCl_3_/300 MHz): δ 1.25–1.35 (m, 8H), δ 1.61–1.71 (m, 4H), 2.52 (s, 3H), 2.64–2.72 (m, 4H), 3.24 (t, *J* = 6.9 Hz, 2H), 4.53 (t, *J* = 6.9 Hz, 2H), 6.96–7.06 (m, 4H), 7.31 (s, 1H); ^13^C-NMR (CDCl_3_/75 MHz): 21.9, 25.6, 28.4, 29.1, 29.3, 29.4, 29.7, 32.7, 38.3, 39.1, 49.1, 121.2, 123.9, 124.3, 130.8, 131.5, 135.9, 137.5, 138.3, 141.5, 148.3. HRMS (ESI^+^) calcd for [C_42_H_56_N_6_S_10_+H]^+^: 965.1846, found 965.1847.

*1,2-Bis(8-(1-(3-(5'-(methylthio)-2,2'-bithiophen-5-ylthio)propyl)-1H-1,2,3-triazol-4-yl)octyl)disulfide* (**12b**). Yield 48%; pale yellow solid; C_44_H_60_N_6_S_10_; mp: 119–121 °C; IR (KBr): 790, 863, 1051, 1123, 1213, 1328, 1426, 1465, 1497, 1551, 2849, 2919, 3062, 3113 cm^−1^; ^1^H-NMR (CDCl_3_/500 MHz): δ 1.28–1.39 (m, 8H), 1.64–1.70 (m, 4H), 2.19–2.25 (m, 2H), 2.51 (s, 3H), 2.65–2.72 (m, 4H), 2.76 (t, *J* = 7 Hz, 2H), 4.47 (t, *J* = 6.5 Hz, 2H), 6.96–7.03 (m, 4H), 7.24 (s, 1H); ^13^C-NMR (CDCl_3_/125 MHz): δ 18.5, 22.1, 25.7, 28.5, 29.2, 29.3, 29.5, 29.6, 35.4, 39.2, 48.2, 58.6, 120.9, 123.9, 124.2, 131.6, 132.3, 135.2, 137.3, 138.6, 141.0, 148.6. HRMS (ESI^+^): calcd for [C_44_H_60_N_6_S_10_+H]^+^: 993.2160, found 993.2165.

## 4. Conclusions

In summary, two series of triazolobithiophenes exhibiting either N–C (compounds **2a-c**) or C–C connections (compounds **3a**,**b**) to the aliphatic side chain were synthesized. Each group contained derivatives of varying spacer length (C_n_, n = 1, 2, 3 carbons) between the triazole and the bithiophene moiety. Except for **2a** and **11a**, all the thiols or their corresponding disulfides yielded stable monolayers with high surface coverage. Due to their excellent durability, the new molecules represent ideal candidates for thorough studies on the energy transmitting principle in DIAMS MS [55]. Consequently, the click chemistry appeared to be suitable for the design of new SAMs bearing a 1,2,3-triazole and various chromophors that exhibit, as the pioneering bithiophene **1**, an absorption maximum which is close to the emission wavelength of a standard nitrogen laser.

## Supplementary Materials

Supplementary materials can be accessed at http://www.mdpi.com/1420-3049/16/10/8758/s1.
