# Supplementary material for: Triazolobithiophene Light Absorbing Self-Assembled Monolayers: Synthesis and Mass Spectrometry Applications
Source: Molecules. 2011 Oct 19;16(10):8758–74. doi: 10.3390/molecules16108758 (PMC6264341; doi:10.3390/molecules16108758)
Supplement: Supplementary file 1 [file molecules-16-08758-s001.doc]

**Supplementary Information**

**Figure 1.** Optical absorption spectrum of **4a** (1.10^−4^ M in CH_2_Cl_2_).

**Figure 2.** Optical absorption spectrum of **4b** (1.10^−4^ M in CH_2_Cl_2_).

**Figure 3.** Optical absorption spectrum of **4c** (1.10^−4^ M in CH_2_Cl_2_).

**Figure 4.** Optical absorption spectrum of **5a** (1.10^-4^ M in CH_2_Cl_2_).

**Figure 5.** Optical absorption spectrum of **5b** (1.10^−4^ M in CH_2_Cl_2_).
